# Hyperelastic Modeling and Validation of Hybrid-Actuated Soft Robot with Pressure-Stiffening

**DOI:** 10.3390/mi14050900

**Published:** 2023-04-22

**Authors:** Majid Roshanfar, Salar Taki, Amir Sayadi, Renzo Cecere, Javad Dargahi, Amir Hooshiar

**Affiliations:** 1 Mechanical Engineering Department, Concordia University, Montreal, QC H3G 1M8, Canada; m_roshan@encs.concordia.ca (M.R.); sa_taki@encs.concordia.ca (S.T.); dargahi@encs.concordia.ca (J.D.); 2 Department of Surgery, McGill University, Montreal, QC H3A 0G4, Canada; amir.sayadi@mail.mcgill.ca (A.S.); renzo.cecere@muhc.mcgill.ca (R.C.)

**Keywords:** soft robot, stiffness, Cosserat rod model, hybrid actuation, tendon-driven, hyperelastic material model, intraluminal applications, pressure-stiffening

## Abstract

Soft robots have gained popularity, especially in intraluminal applications, because their soft bodies make them safer for surgical interventions than flexures with rigid backbones. This study investigates a pressure-regulating stiffness tendon-driven soft robot and provides a continuum mechanics model for it towards using that in adaptive stiffness applications. To this end, first, a central single-chamber pneumatic and tri-tendon-driven soft robot was designed and fabricated. Afterward, the classic Cosserat’s rod model was adopted and augmented with the hyperelastic material model. The model was then formulated as a boundary-value problem and was solved using the shooting method. To identify the pressure-stiffening effect, a parameter-identification problem was formulated to identify the relationship between the flexural rigidity of the soft robot and internal pressure. The flexural rigidity of the robot at various pressures was optimized to match theoretical deformation and experiments. The theoretical findings of arbitrary pressures were then compared with the experiment for validation. The internal chamber pressure was in the range of 0 to 40 kPa and the tendon tensions were in the range of 0 to 3 N. The theoretical and experimental findings were in fair agreement for tip displacement with a maximum error of 6.40% of the flexure’s length.

## 1. Introduction

### 1.1. Background

**Figure 1:**
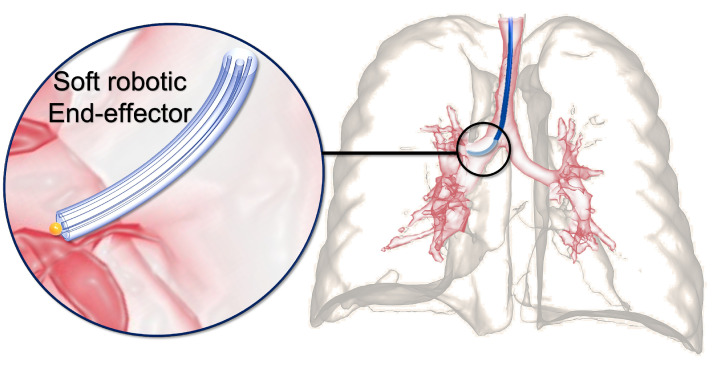
A representative use case of soft robots for intraluminal procedures.

Soft robots have become increasingly prevalent in minimally invasive surgery (MIS) [1] due to their flexible and compliant structural materials, such as elastomers, which make them better suited for interacting with the human body and reducing the risk of injury to surrounding tissue during surgical interventions. Soft robots are highly compatible with human–robot interaction and are ideal for performing procedures through natural orifices or small surgical incisions [2]. Moreover, soft robots can be equipped with soft sensors to provide feedback on the surgical environment, enhancing surgical precision [3,4,5]. Soft robot-assisted MIS systems have been proposed for various applications, including ablation [6], cardiovascular [7], and bronchoscopy procedures [8]. Figure 1 shows the use of a soft surgical robot for intra-bronchial interventions as a representative use case. In this case, a soft robot can navigate through the complex and narrow airways of the lungs to perform diagnostic and therapeutic procedures, such as biopsy and tumor removal. The soft robot’s compliant and flexible structure allows it to adapt to the changing anatomy of the airways, reducing the risk of tissue damage and improving patient outcomes.

In MIS, instruments with rigid end-effectors [9] have been shown to have limitations, such as lack of adaptability leading to over-steering and vessel rupture [7]. Reports have shown that excessive force from rigid instruments can result in embolization, perforation, thrombosis, and dissection of the vascular wall, indicating the need for alternative instruments. Soft robots with hybrid actuation using air pressure and tendons have been proposed as an alternative to rigid instruments, particularly in situations where they are unable to complete the surgical task or access unreachable points [10,11]. These soft instruments offer increased safety during the intervention and can facilitate faster recovery after surgery [12]. Various researchers have investigated the development of soft surgical instruments, such as those with antagonistic actuation [13], stiffness control, and capability to adapt to the surgical environment, to improve surgical outcomes.

### 1.2. Related Studies

Soft robots are continuum robots that, in theory, have an infinite number of Degrees of Freedom (DoF), but in practice, they are controlled by a limited number of kinematic inputs [14]. It is important to note that these robots are vulnerable to a decrease in their ability to exert force on the environment along their length [15]. To measure the deformation of soft robots, researchers have employed Euler-Bernoulli’s beam theory [16,17], which simplifies the mechanical modeling process. However, it has been experimentally discovered that the relationship between bending moment and deformation is highly nonlinear, indicating that this theory is no longer valid for the extreme loading conditions of soft robots [18].

Another approach to modeling soft robots involves using constant or variable curvature models [19], or the widely used Piece-wise Constant Curvature (PCC) model [20]. However, these models fail to adequately capture pressure-stiffening phenomena such as the effect of shear, leading to inaccuracies. Although integrating Finite Element Analysis (FEA) with PCC can enhance the kinematic model of soft robots [21], this remains a computationally expensive approach, limiting its practicality for real-time applications. As an alternative, recent literature has suggested modeling soft robots as one-dimensional slender objects based on the Cosserat rod model [22,23]. This approach allows the effects of gravity, torsion, and external loads to be incorporated and simplifies the model derivation process [10], treating small and large deformations of soft robots with a unified formulation. Research has shown that the Cosserat rod model outperforms the PCC model when shear and gravitational loading are considered [24], and its ability to incorporate hyperelasticity of materials enables accurate tracking of pressure-stiffening phenomena. Table 1 summarizes recent research on modeling the multi-physical deformation of soft-actuated robots, highlighting the Cosserat rod model’s superiority in capturing highly nonlinear deformation behavior under various loading conditions. This ability distinguishes it from other models that neglect material nonlinearity and results in a more precise representation of the mechanical response of soft robots. For a comprehensive study on the modeling of continuum robots, see [25,26].

In the current study, a novel mechanistic model based on the Cosserat rod theory is proposed to investigate the effect of tendon forces and pneumatic chamber pressure on the pressure-stiffening of a soft robot. The Cosserat rod model is a powerful tool for modeling the highly nonlinear deformation behavior of soft robots and can capture the pressure-stiffening phenomenon through a hyperelastic constitutive model. However, it is crucial to experimentally characterize this effect and validate the model’s accuracy. Therefore, the proposed Cosserat rod model was validated using a soft robot with three tendons and one central air pressure chamber. The pressure-stiffening phenomenon is essential for soft robots’ functionality and control, and accurately modeling it is critical for the development of advanced soft instruments for robot-assisted surgical interventions. The proposed model’s validation against a real soft robot demonstrates its potential for accurately capturing the complex mechanical behavior of soft robots under various loading conditions. Furthermore, this study highlights the importance of combining experimental validation with theoretical modeling to ensure the accuracy and reliability of the proposed model.

### 1.3. Contributions

A key contribution of this study was modeling and experimentally validating a tendon-driven pneumatically actuated soft robot with a pressure-stiffening effect. One major difference between this work and previous studies is the incorporation of hyperelastic effects and stiffness adaptation using auxiliary pressure. Although previous studies have focused on modeling and experimenting with soft robots, our approach advances the understanding of how soft robots behave under different conditions by taking into account their nonlinear material properties. By incorporating hyperelasticity and stiffness adaptation using an auxiliary pressure, our model can better capture the pressure-stiffening effect and adapt its flexural rigidity. The soft robot has various inputs, i.e., air pressure and tendons tension. Therefore, changing the soft robot’s air pressure and tendons’ tension will change its deformability during the intervention. To be more specific, the main contributions of the study were:

1. Developing a Cosserat rod model to account for pressure-stiffening phenomena in a soft robot under both pneumatic and tendon-driven modalities by considering the tangent elastic modulus as a function of internal pressure,
2. Integrating the hyperelastic constitutive model into the Cosserat rod framework to accurately capture the material nonlinearity,
3. Experimental validation of the proposed model for capturing the effects of pressure-stiffening effect during hybrid tendon-pneumatic actuation,
4. Predicting the adaptive flexural rigidity of an inflatable tendon-driven soft robot by using a nonlinear constitutive law in a Cosserat rod framework.

This study represents a significant improvement over previous work, as it can predict the adaptive flexural rigidity of an inflatable tendon-driven soft robot by incorporating a nonlinear constitutive law into a Cosserat rod framework. Although earlier studies have mainly focused on modeling and experimental analysis of soft robots, this study expands the understanding of soft robot behavior by accounting for their nonlinear material properties. Notably, the maximum error was reduced in this study by relating the material property of the soft robot to the chamber pressure, which was a limitation in previous work where the material property remained constant during inflation. In addition, the experimental section of this study evaluated the soft robot’s performance in 3D space, which provides a more comprehensive analysis compared to previous studies that only tested 2D bending. The use of an electromagnetic tracking sensor at the tip of the soft robot enabled the capture of its 3D deformation, as opposed to a camera that was positioned perpendicular to the bending plane and used to measure the bending angle in 2D in the previous study.

## 2. Materials and Methods

### 2.1. Mechanistic Modeling

#### 2.1.1. Kinematics

The kinematic equations are important for understanding the overall behavior of the soft robot and how it responds to external stimuli. By knowing the internal strains throughout the length of the robot, one can determine the amount of force required to achieve a particular deformation or how the robot will respond to a specific loading condition. Soft robots can be described mathematically using a set of differential equations derived from the nonlinear Cosserat rod theory. Typically, soft robots are modeled as flexible and slender objects. This study makes certain assumptions, such as the impact of the robot’s internal chamber pressure on the deformation of its cross-sectional area is assumed to be insignificant. Additional assumption is the constancy of tension forces along the tendon’s length, and the exclusion of viscoelastic properties and rate-dependent behavior (e.g., creep and relaxation) in the two-term Mooney–Rivlin (2MR) material model. The information presented is derived from relevant research works cited in [11,34]. Due to its weight, the soft robot was subjected to distributed gravitational forces. To eliminate the effect of this force on the initial posture of the robot, it was placed upside down throughout the duration of the experiment.

**Figure 2:**
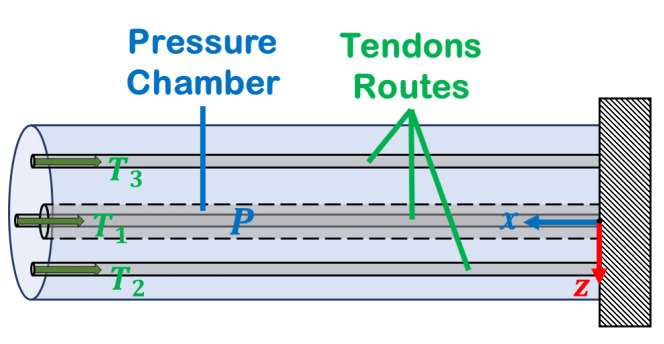
Schematic initial shape of the soft robot with central pressure *P* and three tendon forces $T_{i}$.

**Figure 3:**
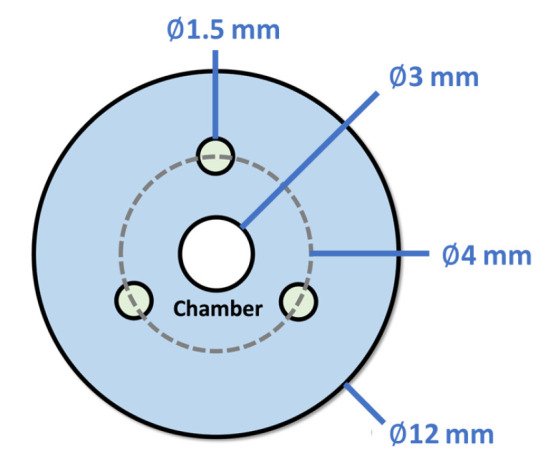
Cross-section of the hybrid-actuated soft robot.

Figure 2 shows a cantilever hollow soft robot with a cross-sectional area *A* in its initial shape, subjected to an internal pressure *P* and three tendon forces at the tip $T_{i}$. Additionally, the cross-section of the soft robot in Figure 3 shows tendons that are angularly separated by 120${}^{\circ }$, with an equal offset from the center. Each point on the backbone is parameterized by an arc parameter s∈[0L], in which *L* is the initial length of the soft robot, and a locally orthonormal frame R(s) [20]. With the given shape parameterization, the position of any point on the backbone of the soft robot can be determined relative to the base of the arc at a distance *s* using the position vector p(s). The partial derivative of p(s) with respect to the arc length in the local frame yields the extension and shear strains along the backbone, denoted by v(s). This strain vector v(s) can be further classified as the “linear strain” of the soft robot, as described in [22] as follow:

(1) $$v\left(s\right)=R^{T}\left(s\right)\frac{\partial p(s)}{\partial s},$$

the “angular strain” vector, denoted by u(s), is represented by the partial derivative of R(s) over the arc length in the local frame. This term is responsible for the curvature due to bending and torsion strains. In other words, u(s) captures the deformation of the soft robot’s backbone in terms of its angular changes along the arc length [22].

(2) $$u\left(s\right)={\left(R^{T}\left(s\right)\frac{\partial R(s)}{\partial s}\right)}^{\vee },$$

where ${\left(.\right)}^{\vee }$ is the vee-operator, a mapping for so(3) to $R^{3}$ [35]. The kinematic equations describe the relationship between the internal strains and the overall shape of the soft robot by representing its geometry as a parameterized curve. These equations provide a way to calculate the local curvature and twist of the robot’s backbone and the rotation of its cross-section.

#### 2.1.2. Force Balance

The Cosserat rod theory as presented in [22] was utilized to derive the quasi-static balance equations of the soft robot. It was achieved by eliminating the time derivative from the dynamic equations. This assumption is reasonable given the low velocity and inertia of the system during intervention, making any temporal variation negligible. The quasi-static balance equations are as follows:

(3) $$\begin{array}{cc} \frac{\partial p(s)}{\partial s} & =R(s)v(s), \end{array}$$

(4) $$\begin{array}{cc} \frac{\partial R(s)}{\partial s} & =R\left(s\right){\left(u\left(s\right)\right)}^{\wedge }, \end{array}$$

(5) $$\begin{array}{cc} \frac{\partial n(s)}{\partial s} & =-\rho Ag-P\frac{\partial R}{\partial s}A^{ch}{\hat{e}}_{1}-T^{t}, \end{array}$$

(6) $$\begin{array}{cc} \frac{\partial m(s)}{\partial s} & =-{\left(\frac{\partial p(s)}{\partial s}\right)}^{\wedge }n\left(s\right)-PA^{ch}R\left(v\times {\hat{e}}_{1}\right)-l^{t}. \end{array}$$

where n(s) and m(s) are the internal force and moment vectors in the global coordination system, ρ is the mass density (constant), $A^{ch}$ is the cross-sectional area of the air chamber, g is the gravity vector, ${\hat{e}}_{1}$ is the unit vector, ${\left(.\right)}^{\wedge }$ is the hat-operator, a mapping from $R^{3}$ to so(3) [35]. From a mechanical perspective, the chamber pressure causes a uniform longitudinal tensile force along the entire length of the soft robot. This force has a magnitude of PA, where *A* represents the cross-sectional area of the robot that is perpendicular to its backbone. Notably, the internal pressure displays symmetry about the longitudinal axis of the robot, and therefore does not have any impact on the distribution of forces in planes that are perpendicular to the axis. Moreover, $T^{t}$ and $l^{t}$ are the tendons forces and moments, respectively, and in terms of backbone kinematic parameters they are defined as [36]:

(7) $$\begin{array}{cc} T^{t} & =R(a+Av_{,s}+Gu_{,s}),\\ l^{t} & =R(b+G^{T}v_{,s}+Hu_{,s}). \end{array}$$

in which:

(8) $$\begin{array}{cc} a & =\sum_{i=1}^{m}a_{i},\;a_{i}=A_{i}\left[\hat{u}\left(p_{i,s}^{b}+r_{i,s}^{t}\right)+r_{i,ss}^{t}\right],\\ b & =\sum_{i=1}^{m}b_{i},\;b_{i}={\hat{r}}_{i}^{t}a_{i},\\ A & =\sum_{i=1}^{m}A_{i},\;A_{i}=-\tau_{i}\frac{{\left({\left(p_{i,s}^{b}\right)}^{\wedge }\right)}^{2}}{∥p_{i,s}^{b}{∥}^{3}},\\ G & =\sum_{i=1}^{m}G_{i},\;G_{i}=-A_{i}{\hat{r}}_{i}^{t},\\ H & =\sum_{i=1}^{m}{\hat{r}}_{i}^{t}G_{i},\\ p_{i,s}^{b} & =\hat{u}r_{i}^{t}+r_{i,s}^{t}+v. \end{array}$$

where $r^{t}$ is the tendon’s offset from the cross-section, $p^{b}$ is the representation of the variable p in the local frame (i.e., $p^{b}=R^{T}p)$, and $\tau_{i}$ is the tendon tension. Equations (1)–(6) describe the nonlinear state-space representation of the soft robot’s mechanics with six state variables, i.e., $\left(\begin{array}{cccccc} v(s) & u(s) & p(s) & R(s) & n(s) & m(s) \end{array}\right)$. The quasi-statics system governed by Equations (1)–(6) have independent variables v(s) and u(s). Hence, in order to relate the external forces acting on the soft robot to its internal loading, it is necessary to implement a material constitutive law. One of the most widely used material constitutive laws is linear elasticity theory. By combining the Cosserat rod model, which serves as the mechanical model, with the appropriate constitutive laws, a set of differential equations can be derived to govern the quasi-static response of the system.

Usually, hyperelastic materials exhibit changes in their mechanical characteristics when subjected to local stretches. Nonetheless, at a specific stretch value at any given (*s*), the tangent moduli $K_{se}$ and $K_{bt}$ depict the mechanical stiffness for unit length. The “se” subscript refers to shear and extension, and “bt” refers to bending and torsion. During the deformation, the tangent elastic modulus of the soft robots can be estimated using the strain energy density function *W* which will be define in the Section 2.2. The basic linear elastic constitutive equations are [22]:

(9) $$\begin{array}{cc} n(s) & =R\left(s\right)K_{se}\left(v\left(s\right)-v^{★}\left(s\right)\right), \end{array}$$

(10) $$\begin{array}{cc} m(s) & =R\left(s\right)K_{bt}\left(u\left(s\right)-u^{★}\left(s\right)\right). \end{array}$$

where ${\left(.\right)}^{★}$ refers to the state variables before deformation (initial state). Assuming, an initially straight soft robot extended along the global x−axis, $v^{★}\left(s\right)={\left(\begin{array}{ccc} 1 & 0 & 0 \end{array}\right)}^{T}$ and $u^{★}\left(s\right)=0$. Additionally, substituting the derived shear and Hooke’s moduli, the tangent stiffness matrices were obtained as:

(11) $$\begin{array}{cc} K_{se} & =\mathrm{diag}\left(\begin{array}{ccc} E\left(P\right)A_{\circ } & G\left(P\right)A_{\circ } & G\left(P\right)A_{\circ } \end{array}\right), \end{array}$$

(12) $$\begin{array}{cc} K_{bt} & =\mathrm{diag}\left(\begin{array}{ccc} G\left(P\right)I_{11} & E\left(P\right)I_{22} & E\left(P\right)I_{33} \end{array}\right). \end{array}$$

with $I_{ii}$ denoting the second moment of inertia about the i=1,2,3 normal bases of the local coordinate systems along the robot, *E* is Young’s modulus which relates to *P*, *G* is the shear modulus, and $A_{\circ }$ the undeformed cross-sectional area of the soft robot.

### 2.2. Constitutive Model

The mechanical behavior of silicone rubber subjected to quasi-static loading is represented through a nonlinear elastic, isotropic, and incompressible model. Hence, ϑ which is the Poisson’s ratio is ≈0.5 for near-incompressible elastomers. To model the hyperelastic constitutive behavior of the soft robot, a 2MR model is utilized. In accordance with the 2MR model, the strain energy density function (*W*) is expressed as:

(13) $$W=C_{10}\left(I_{1}-3\right)+C_{01}\left(I_{2}-3\right),$$

where $C_{10}$ and $C_{01}$ are material constants and $I_{1}$ and $I_{2}$ are the stretch invariants as:

(14) $$\begin{array}{cc} I_{1} & =\lambda_{1}^{2}+\lambda_{2}^{2}+\lambda_{3}^{2},\\ I_{2} & =\lambda_{1}^{2}\lambda_{2}^{2}+\lambda_{2}^{2}\lambda_{3}^{2}+\lambda_{1}^{2}\lambda_{2}^{2},\\ I_{3} & =\lambda_{1}^{2}\lambda_{2}^{2}\lambda_{3}^{2}, \end{array}$$

where $\lambda_{i}$ (i=1,2,3) represents the deformation of a differential cubic volume element along the principal axes of a Cartesian coordinate system and $I_{3}=1$ for an incompressible material such as elastomer in this study. From finite-strain theory in continuum mechanics, $\lambda_{i}$ (i=1,2,3) are the principal stretches that are in tandem square roots of the eigenvalues of the right Cauchy-Green strain tensor C.

### 2.3. Boundary Conditions

It is assumed that the tendon is terminated at the distal end of the soft robot, i.e., s=L, and the tendon tension is applied tangent to the extension direction of the soft robot, i.e., local x−direction, thus:

(15) $$\begin{array}{cc} n{|}_{s=L} & =n_{i}^{t}{|}_{s=L}+n^{pr}{|}_{s=L}=-\tau_{i}\frac{Rp_{i,s}^{b}\left(L\right)}{∥Rp_{i,s}^{b}\left(L\right)∥}+PA^{ch}R{\hat{e}}_{1}, \end{array}$$

(16) $$\begin{array}{cc} m{|}_{s=L} & ={\hat{Rr}}_{i}^{t}n_{i}^{t}{|}_{s=L}+l_{pr}{|}_{s=L}=-{\hat{Rr}}_{i}^{t}\tau_{i}\frac{Rp_{i,s}^{b}\left(L\right)}{∥Rp_{i,s}^{b}\left(L\right)∥}+PA^{ch}R\left[v\times {\hat{e}}_{1}\right]. \end{array}$$

Also, the initial set of boundary conditions (BCs) relates to the kinematic constraints at the base of the soft robot, i.e., at s=0. From a mechanical standpoint, the soft robot can be considered as a cantilever, thereby implying that:

(17) $$\begin{array}{cc} p{|}_{s=0} & =p_{0}, \end{array}$$

(18) $$\begin{array}{cc} R{|}_{s=0} & =I_{3\times 3}, \end{array}$$

where $p_{0}$ is the original shape of the soft robot before deformation. These BCs translate into six independent scalar kinematic constraints. The set of six boundary conditions described here are six distinct and independent conditions that the solution must satisfy. These boundary conditions specify particular constraints that the solution must meet at both ends of the soft robot. Meeting these conditions creates a boundary-value problem (BVP), which involves finding a solution to a set of differential equations that satisfies the specified constraints at the boundaries.

### 2.4. Solution Schema

In order to determine the deformation of the soft robot, the constitutive equations were first inserted into the force equations. It is presumed that the initial posture of the robot is entirely known, thus enabling the formulation of an accurate mathematical model to assess its mechanical response. For the system of nonlinear differential equations (Equations (1)–(6)), there are two BCs for n and m at the distal end, and others concerning to p, R available at the proximal end of soft robot. The problem was tackled using a shooting method, which involved beginning a simulation loop and iteratively solving sets of equations along with their corresponding boundary conditions. This process eventually resulted in visualizing the system’s response.

### 2.5. Pressure-Stiffening and Tangent Modulus

The tangent moduli, $K_{se}\left(P\right)$ and $K_{bt}\left(P\right)$ were determined as functions of *P* using the 2MR model and continuum balance equations. Based on the definitions in (1), $v-v^{★}$ is the Lagrangian finite strain for shear and extension modes and $u-u^{★}$ is the Lagrangian finite strain for bending and torsion. Assembling the Lagrangian finite strain Y with adding local strains based on the prescribed internal forces and moments from extension, shear, and bending in tangent and oscular local planes resulted in:

(19) $$Y=\left(\begin{array}{ccc} v_{1} & v_{2}+u_{1} & v_{3}-u_{1}\\ v_{2}+u_{1} & -\vartheta v_{1} & 0\\ v_{3}-u_{1} & 0 & -\vartheta v_{1} \end{array}\right),$$

with ϑ≈0.5 denoting the Poisson’s ratio for incompressible polymers. On the other hand, the Lagrangian strain tensor is related to the right Cauchy-Green deformation tensor C such that:

(20) C=2Y+I,

with $I_{ij}=\delta_{ij}$, the Kronecker’s delta. On the other hand, the first and second principal invariants can be calculated from C using:

(21) $$\begin{array}{cc} I_{1} & =\mathrm{Tr}(C), \end{array}$$

(22) $$\begin{array}{cc} I_{2} & =\frac{1}{2}\left(\mathrm{Tr}{\left(C\right)}^{2}-\mathrm{Tr}\left(C^{2}\right)\right), \end{array}$$

(23) $$\begin{array}{cc} I_{3} & =\det(C)=1. \end{array}$$

where Tr$\left(X_{ij}\right)=X_{ii}$ is the trace operator (Einstein notation). Afterward, the second Piola Kirschoff stress σ definition in terms of C was used to find the relationship between u and v with stress components:

(24) $$\sigma \left(P,C\right)=-p^{★}I_{3\times 3}+2C_{10}C^{T}-2C_{01}C^{-T}.$$

with volumetric stress component $p^{★}$:

(25) $$p^{★}=\frac{2}{3}\left(C_{10}I_{1}-C_{01}I_{2}\right).$$

As can be seen in (24), the stress throughout the robot’s body is affected by the internal pressure’s effect on strain components manifested in σ while the robot’s material properties affect the stress through $C_{01}$ and $C_{10}$. In the end, to obtain the tangent elastic modulus the $\sigma_{11}$ component of the stress tensor was used to obtain *E* as a function of *P*:

(26) $$E\left(P\right)=\frac{\partial \sigma_{11}}{\partial \lambda_{1}}=\frac{\partial \sigma_{11}}{\partial I_{1}}\frac{\partial I_{1}}{\partial \lambda_{1}}+\frac{\partial \sigma_{11}}{\partial I_{2}}\frac{\partial I_{2}}{\partial \lambda_{1}},$$

where $\lambda_{1}$ is the square root of the largest eigenvalue of *C* tensor.

To illustrate the effect of pressure on tangent elastic modulus three load cases were simulated. The first load case was pressurizing the chamber in the absence of tendon tension. This load case would theoretically result in mere tension in the soft robot and elongate its length without a significant bending effect. The second load case was applying a 3 N tensile force to one of the tendons in the absence of chamber pressurization. Theoretically, it was not expected to observe significant pressure-stiffening. However, small changes in elastic modulus were expected due to the natural strain-stiffening of the hyperelastic material. The third load case was the combination of load case 1 and load case 2, where the chamber was pressurized gradually up to 40 kPa in the presence of a 3 N tendon tension.

## 3. Validation Study

### 3.1. Soft Robot Design

In this study, a soft robot made of Ecoflex 00-50 with a central chamber and three tendons is designed. The outer diameter of the soft robot was 12 mm, and its length was 85 mm. In this design, the central chamber diameter was 3 mm, the chamber length was 80 mm, the diameter of tendon passages was 1.5 mm, and the tendons’ offset is 4 mm. Table 2 summarizes the model parameters and 2MR material constants [37] used in the study.

### 3.2. Experimental Setup

**Figure 4:**
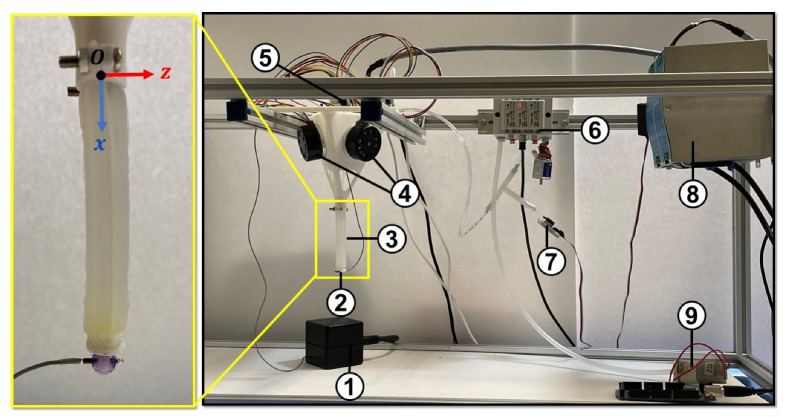
Components of the mechanical and electrical modules in the prototyped soft robot (1) source of electromagnetic field (2) tracking sensor (3) soft robot (4) motors (5) motor controller (6) electronic pressure controller and manifold (7) pressure sensor (8) power supply (9) air pump.

To construct the soft robot, a cylindrical mold was initially created using a 3D printer (Replicator+, MakerBot, New York, NY, USA) and PLA material. This mold featured internal channels for the tendons and chamber. Additionally, a housing platform was 3D-printed to attach the soft robot’s base to the aluminum frame. The soft robot’s body was made using Ecoflex 00-50 (Smooth-On Inc., Macungie, PA, USA), which was produced by mixing parts A and B (50:50) and degassing the silicone mixture in a vacuum chamber. After resting for 24 h at 24 °C for curing, the soft robot was ready for testing. Figure 4 depicts the experimental setup used in this study. An air pump (KPM27CKoge Electronics, KMP Electronics Ltd., Sofia, Bulgaria) was used to supply air pressure, while a pressure sensor (Phidgets Inc., Calgary, AB, Canada) was used to record the chamber’s pressure in real time during the experiment. An electronic pressure regulator (ITV0010-3UML, SMC, Tokyo, Japan) was employed to set the internal pressure of the soft robot. To track the soft robot’s tip position, an electromagnetic motion tracker (Microsensor 1.8${}^{\mathrm{TM}}$, Polhemus, Colchester, VT, USA) was used. Three motors (Maxon, EC 45 flat, 60 W, Irvine, CA, USA) with a digital positioning controller (Maxon, EPOS4 Compact, Irvine, CA, USA) were also integrated into the setup to provide tendon force at the tip of the soft robot. They were connected to a power supply (24 V, 10 A).

**Figure 5:**
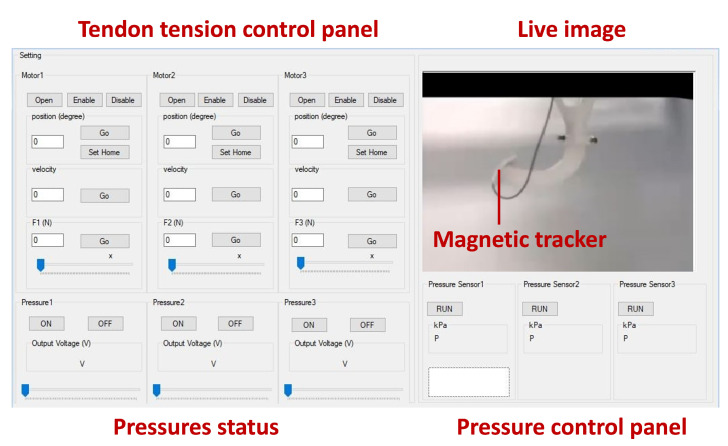
Tendon tension and pressure control panel of the experimental setup.

Additionally, dedicated software was developed in C# programming language for data acquisition and control of pneumatic servo-valves and motor torque control. Motors’ torque control was performed using Maxon’s software development kit and the motor driver’s internal proportional-integral controller. Using the pulley’s diameter, the tendon tension was estimated. Figure 5 shows the control panel of the setup.

### 3.3. Study Protocol

**Figure 6:**
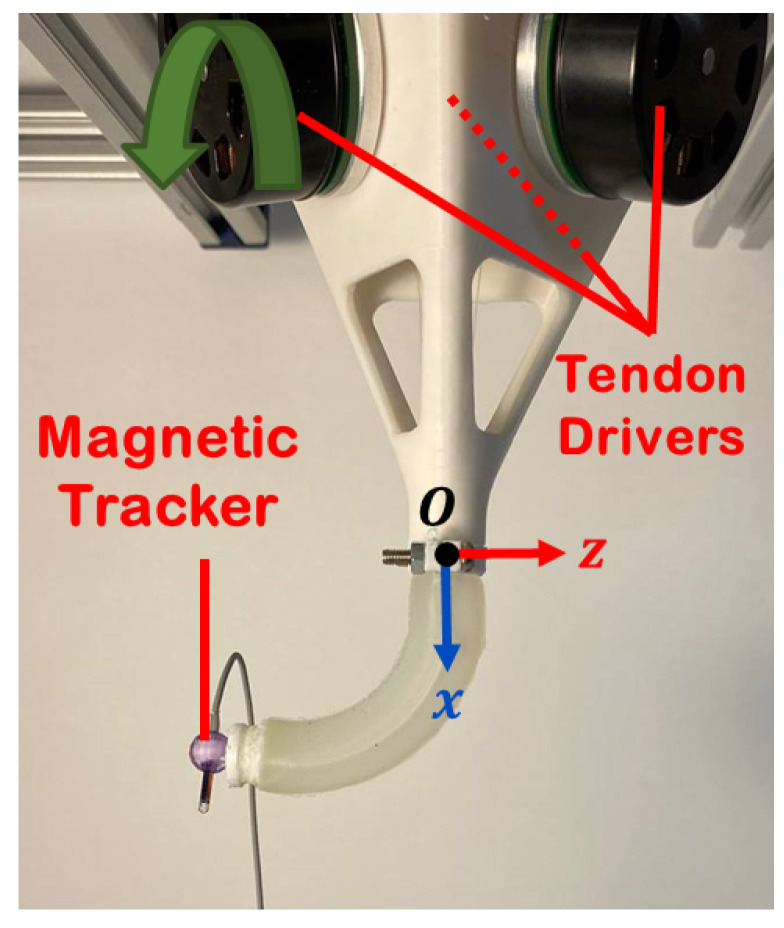
The deformation of a soft robot under the force of a 3 N tendon tension without the presence of internal chamber pressure.

The aim of conducting the experiments is to confirm the validity of the hybrid-actuated soft robot model that has been proposed based on the Cosserat rod model. As part of the validation study, 15 experiments were conducted, i.e., with varying chamber pressures in the range of 0 to 40 kPa as well as varying tendon forces in the range of 0 to 3 N. To increase the reliability of the data acquisition, each experiment was repeated three times, and the average of the results is reported. Each time a fixed internal pressure was applied, then the tendon forces increased, and the soft robot began to deform from its resting position. The first tendon force gradually increased from 0 to 3 N in each experiment while the pressure inside the air chamber remained constant. Then, the first tendon released upon the tip of the soft robot returns to the initial position. The second and third tendons are applied in the same manner. The experiment was again repeated for the internal chamber pressure to be 10, 20, 30, and 40 kPa. When the first tendon was pulled, the tip of the soft robot moved in the y-direction and the distance of the tip in the x-direction was reduced. During the pulling of tendon 2, its displacement increased in the z-direction and decreased in the y-and x-directions. In parallel, a magnetic tracking sensor saved the tip position of the soft robot for validation comparison. Using the recorded current data from the motors, the developed model was solved, and the computed tip positions were compared with the ground truth. Figure 6 shows the tip trajectory of a soft robot under the force of a 3 N tendon tension without the presence of internal chamber pressure *P* = 10 kPa.

### 3.4. Results and Discussion

**Figure 7:**
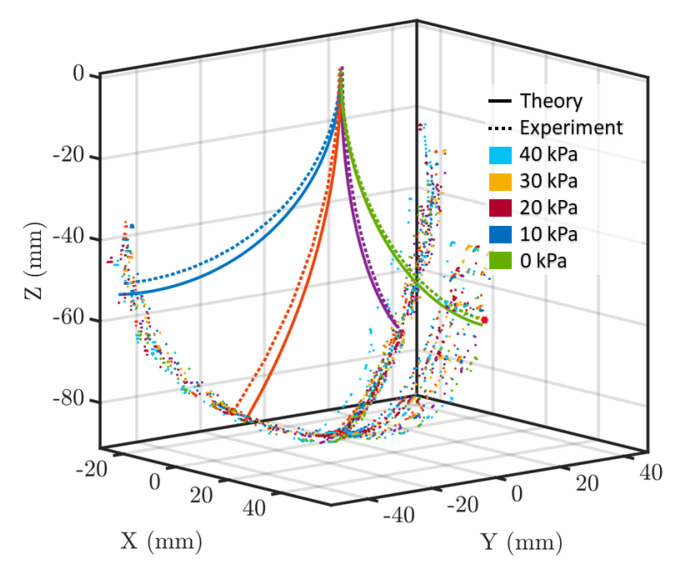
Comparison of the proposed Cosserat model with experimental setups under various internal pressures.

Based on the results of the experimental study, it was shown that, in accordance with the theories, for a given tendon tension and internal chamber pressure, the soft robot would have a pressure-stiffening effect. Figure 7 and Table 3 demonstrates the error of tip displacement in each experiment.

**Figure 8:**
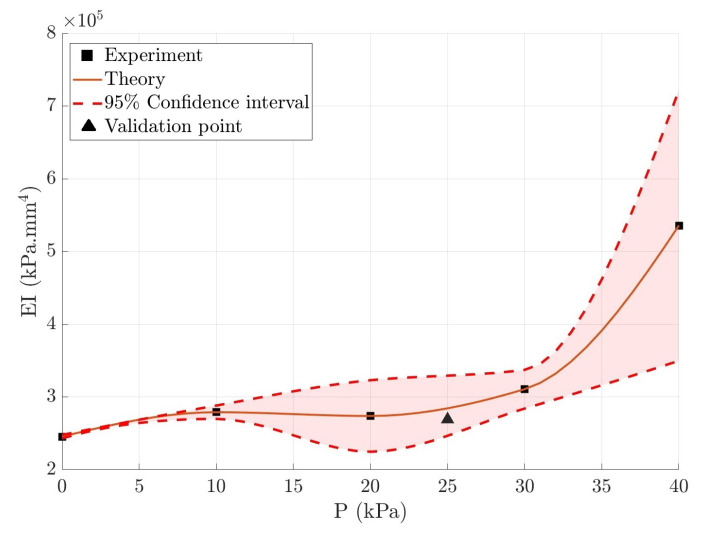
Variation of flexural rigidity of the soft robot with chamber pressure pressure-stiffening.

Figure 8 shows the variation in rigidity (EI) of the soft robot with respect to the internal chamber pressure. This experiment is repeated three times for each chamber pressure, and the solid line indicates the average value. As the figure illustrates, increasing the internal pressure leads to higher rigidity. The variation in rigidity follows the hyperelastic model of the material used to model the soft robot. As the air pressure inside the chamber increases, two characteristics are playing a role in increasing the EI. The first is the E(P), which will increase due to the strain-stiffening effect. The second factor is the radial expansion of soft robots, which results in an increase in *I*. It was proposed to use the fabric reinforcements to prevent excessive radial expansion to constrain *I* [38]. In addition, a validation point with P=25 kPa was examined to verify the prediction of rigidity. It was found that the point was within the 95% confidence interval. In addition, an optimization method was used to determine the EI to reduce the error between the deformation that was predicted by the Cosserat model and experimental data. To this end, through an iterative process, the found EI is substituted into the constitutive equations to determine the position vector of the soft robot, p(s).

The results of the study indicate that the Cosserat rod model can predict the pressure-stiffening effect of soft robots. It was observed that the theoretical prediction had a maximum relative mean absolute error (MAE) of 6.4% of the robot’s length. It is noteworthy that during the experiment, the tendon tensions were changed in the range of 0 to 3 N causing the tip of the robot to move in 3D space (Figure 6). The observed errors might have been because the viscoelastic behavior of the soft robot was neglected, and the radial expansion of the robot was not taken into account.

## 4. Conclusions

Throughout this study, the proposed mechanistic model of the soft robot was solved to determine a shape for the robot at various pressures and tensions of its tendons. The proposed approach extends upon the current literature by including hyperelastic effects and stiffness adaptation using an auxiliary pressure. To include the effects of pressure-stiffening, the volumetric stress component in the two-term Mooney–Rivlin material model was modified. This modification then leads to an input-dependent tangent stiffness tensor in the Cosserat rod model. The experimental validation confirmed the accuracy of the proposed model for each of the different scenarios. In the future, to evaluate the accuracy of the leader–follower performance of the soft robot, a model-based position controller for the robot will be developed, and the accuracy of tip-tracking will be investigated for simple and tortuous trajectories to assess the performance. Additionally, an extension of this work would be the inclusion of the effect of the ratio of robot cross-sectional area to length on the accuracy of the model. Flexure slenderness might affect the boundaries (of pressure and tendon tension) of the validity of the proposed model. In addition, the feasibility of real-time dynamics of a hybrid-actuated soft robot based on Cosserat rod models will be investigated. It is expected that this feature will increase the state-of-the-art of soft surgical robots by adding a new capability for adaptability during the interventions.

## Figures and Tables

**Table 1:** Comparison of a representative set of recent literature on mechanical modeling soft robots.

Study	Method	Material Model	Hybrid Actuation	Dynamic	Error (mm)
Roshanfar et al. [10]	Cosserat	Non-Linear	√	×	8.25%
Hooshiar et al. [23]	Cosserat	Linear	×	×	7%
Wang et al. [27]	Cosserat	Linear	×	√	-
Dou et al. [28]	Euler-Bernoulli	Linear	√	×	Less than 8%
Huang et al. [29]	Variable Curvature	Linear	×	×	2.89%
Niu et al. [30]	Cosserat	Linear	×	×	Less than 4%
Ghoreishi et al. [31]	Euler-Bernoulli	Linear	×	√	-
Li et al. [32]	Cosserat	Linear	×	√	Less than 5%
Caasenbrood et al. [33]	Piece-wise Constant Curvature	Non-Linear	×	√	RMS error was ±0.55

**Table 2:** Model parameters and 2MR material constants of the prototyped soft robot.

Length	Outer Dia.	Inner Dia.	Density	Tendon Offset	2MR Constants [37]	
*L*	$D_{o}$	$D_{i}$	ρ	*r*	$C_{10}$	$C_{01}$
(mm)	(mm)	(mm)	($\frac{g}{cc}$)	(mm)	(MPa)	(MPa)
85	12	3	1.070	4	0.0188	−0.014

**Table 3:** Cosserat rod model prediction error with respect to experimental observations.

Pressure (kPa)	Tendon Tension (Sequential)			Tip Error Rel. MAE (%)
	F1	F2	F3	
	(N)	(N)	(N)	
0	0–3	0	0	5.58%
10	0–3	0–3	0	5.12%
20	0–3	3	0–3	5.98%
30	0	0–3	0–3	5.89%
40	0	0	0–3	6.40%
Mean:				5.79%

## Data Availability

Data are unavailable.

## Abbreviations

The following abbreviations are used in this manuscript:

MIS	Minimally Invasive Surgery
DoF	Degrees of Freedom
PCC	Piece-wise Constant Curvature
2MR	2-term Mooney–Rivlin
FEA	Finite Element Analysis
BC	Boundary Condition
BVP	Boundary-Value Problem
